# A Novel Lightweight Wearable Soft Exosuit for Reducing the Metabolic Rate and Muscle Fatigue

**DOI:** 10.3390/bios11070215

**Published:** 2021-06-30

**Authors:** Lingxing Chen, Chunjie Chen, Zhuo Wang, Xin Ye, Yida Liu, Xinyu Wu

**Affiliations:** 1 Shenzhen Institute of Adanced Technology, Chinese Academy of Sciences, Shenzhen 518055, China; lx.chen@siat.ac.cn (L.C.); zhuo.wang@siat.ac.cn (Z.W.); xin.ye@siat.ac.cn (X.Y.); yd.liu1@siat.ac.cn (Y.L.); xy.wu@siat.ac.cn (X.W.); 2 Shenzhen College of Advanced Technology, University of Chinese Academy of Sciences, Shenzhen 518055, China; 3 Guangdong Provincial Key Lab of Robotics and Intelligent System, Shenzhen Institute of Advanced Technology, Chinese Academy of Sciences, Shenzhen 518055, China; 4 CAS Key Laboratory of Human-Machine Intelligence-Synergy Systems, Shenzhen Institute of Advanced Technology, Shenzhen 518055, China; 5 Guangdong-Hong Kong-Macao Joint Laboratory of Human-Machine Intelligence-Synergy Systems, Shenzhen 518055, China

**Keywords:** wearable soft exosuit, lightweight, metabolic rate, muscle fatigue, comfort

## Abstract

Wearable robotic devices have been proved to considerably reduce the energy expenditure of human walking. It is not only suitable for healthy people, but also for some patients who require rehabilitation exercises. However, in many cases, the weight of soft exosuits are relatively large, which makes it difficult for the assistant effect of the system to offset the metabolic consumption caused by the extra weight, and the heavy weight will make people uncomfortable. Therefore, reducing the weight of the whole system as much as possible and keeping the soft exosuit output power unchanged, may improve the comfort of users and further reduce the metabolic consumption. In this paper, we show that a novel lightweight soft exosuit which is currently the lightest among all known powered exoskeletons, which assists hip flexion. Indicated from the result of experiment, the novel lightweight soft exosuit reduces the metabolic consumption rate of wearers when walking on the treadmill at 5 km per hour by 11.52% compared with locomotion without the exosuit. Additionally, it can reduce more metabolic consumption than the hip extension assisted (HEA) and hip flexion assisted (HFA) soft exosuit which our team designed previously, which has a large weight. The muscle fatigue experiments show that using the lightweight soft exosuit can also reduce muscle fatigue by about 10.7%, 40.5% and 5.9% for rectus femoris, vastus lateralis and gastrocnemius respectively compared with locomotion without the exosuit. It is demonstrated that decreasing the weight of soft exosuit while maintaining the output almost unchanged can further reduce metabolic consumption and muscle fatigue, and appropriately improve the users’ comfort.

## 1. Introduction

The field of interaction is receiving increasing attention in many application domains [1,2], such as mobility assistance, autonomous driving, robotics industry and medical field [3,4]. More and more robot designers not only value performance indicators, but also begin to focus on maintaining the balance between robots’ performance and users’ comfort, hoping to increase the user experience and comfort while improving performance.

The wearable soft exosuit is an emerging robot with appreciable wearability, and the joint torque generated is also sufficient to enhance the user’s movement and physical capabilities. Recently, development of soft exosuit has made important progress, mainly in fields of rehabilitation, military and industrial [5,6,7]. From the point of view of ordinary users, the soft exosuit has better shape adaptability, lower-key appearance, better compliance, and much lighter weight than traditional exoskeleton, making it more suitable to wear [8,9]. The soft exosuit can also be divided into single-joint assisting and multi-joint combined assisting depending on the number of assisted joints [10,11,12]. According to the power source, it is divided into powered and unpowered exoskeletons, both of which are the research hotspots of exoskeletons. During exercise, the unpowered exoskeleton stores energy such as elastic potential energy to provide assistance for the next moment of exercise [13]. Even an unpowered exoskeleton (which designed by Michael et al.) utilizes the collected energy to generate electricity and reduces metabolic consumption [14]. Compared to powered exoskeletons, unpowered exoskeletons are lighter. However, because there is no external energy input, most unpowered exoskeletons are lower than powered exoskeletons in reducing metabolic consumption. Nowadays, many soft exosuits aim to reduce more metabolic consumption. The general way is to increase the output power of the entire soft exosuit system, which often leads to an increase in the whole system’s weight. However, for some people, such as the elderly and even some people who wear soft exosuit for the first time, they may feel unaccustomed or even feel uncomfortable due to the weight of soft exosuit on the body. Therefore, if the assistance force can be maintained and reduce the mass of soft exosuit as much as possible, it will be possible to improve the comfort of users and further reduce metabolic consumption.

The work in this paper mainly presents a lower limb powered soft exosuit, which is currently the lightest soft exosuit known. From the perspective of user comfort, reducing the weight of the whole system as much as possible under the condition of keeping the output power of the soft exosuit system unchanged will be a suitable method. The experiments in this paper also proved that reducing the weight of the system could not only improve the comfort of users, but also further reduce metabolic consumption.

Wyss Institute for Biologically Inspired Engineering proposed the first generation of lower limbs soft exosuit, which used McKibben pneumatic muscle as actuator, had an epoch-making significance [15] and the whole system had a mass of 7.144 kg. In 2019, Kim et al. presented a versatile and portable exosuit for walking and running, of which weight is 5.0 kg [16], and the exosuit automatically switches between actuation profiles for both gaits, on the basis of estimated potential energy fluctuations of the wearer’s center of mass [16]. In order to optimize control performance, Ye Ding et al. used Bayesian optimization to continuously optimize control parameters suitable for users during walking [17]. Juanjuan Zhang et al. proposed an optimization algorithm, human-in-the-loop (HIL), to simultaneously optimize multiple device characteristics on the basis of measured human parameters [18]. In this paper, more attention is paid to human comfort, and a lightweight soft exosuit is proposed to improve human comfort and reduce metabolic consumption.

In this paper, a novel lightweight soft exosuit is proposed, to improve user comfort. The prototype of soft exosuit will be introduced in Section 2, and then in Section 3, the control method will be explained, and Section 4 will illustrate how to evaluate the performance of the lightweight soft exosuit through the metabolic consumption and muscle fatigue experiments. The discussion between the lightweight soft exosuit and other soft exosuits will show in Section 5. Finally, conclusion will be presented in Section 6.

## 2. System Overview

**Figure 1:**
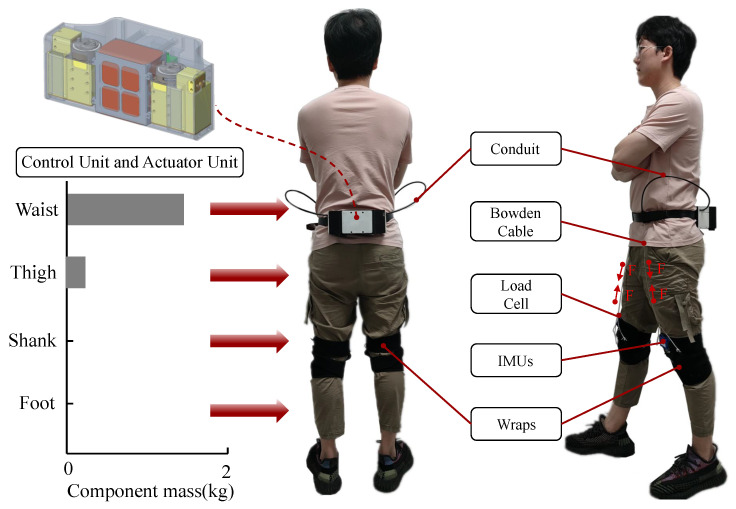
The lower limb lightweight soft exosuit. The whole system is worn on the waist and thighs of humans. The F represents the direction of assistance force.

In this paper, the lower limb lightweight soft exosuit is shown in Figure 1. It can provide assistance for hip joint flexion [19], helping the elderly and patients with rehabilitation training and reducing the consumption of metabolism [20,21].

### 2.1. Prototype of Soft Exosuit

The soft exosuit, as a wearable walking assistance device, is not supposed to hinder people’s normal movement during walking. To achieve this design criteria, we use functional apparel to attach the device to the wearer, with cables that transmit moments in concert with the combined moment that results from different biological muscles [16]. We previously developed an exosuit that can significantly reduce metabolic rate by 6% in flat ground walking, 10% in uphill walking and 14% in stairs climbing tasks on average by assisting lower-limb multi-joint [22]. In the current study, we aim to develop and test a lightweight soft exosuit that assists with hip flexion and reduces the user’s burden caused by the weight of the whole system, to improve human comfort. Although some researches have shown that hip extension assisted (HEA) soft exosuit can reduce more metabolic consumption than hip flexion assisted (HFA) soft exosuit [20]. However, at least 10 volunteers who wear soft exosuit with hip extension assistance and hip flexion assistance respectively, and were invited to participate in the subjective evaluation experiment. Most of the volunteers believe that the hip flexion assisted can give them a more obvious assistance effect than the hip extension assisted during walking. Since the user’s subjective evaluation should also be an important evaluating indicator. In order to make the user more comfortable and reduce metabolism, it is correct to use the hip flexion assisted lightweight soft exosuit as the experimental prototype under the consideration of users’ comfort.

The lower limb soft exosuit has extensive applications, such as enhancing athletic ability and rehabilitation training [10]. As shown in Figure 1, the lightweight soft exosuit is structured mainly with control unit, actuator unit, Bowden cable, inertial measurement unit (IMUs is a kind of biological sensor), load cell, wraps and elastic cable in this paper. The control unit is composed of STM32 control board, bluetooth module and communication module, processes the signals collected by the sensors and sends commands to the actuator unit. The actuator unit includes two motors (DJI 2006) and drives, which tighten the Bowden cable to help the hip joint flexion during walking. The structure of the whole system is compact, and it is made of light-weight aluminum alloy and resin materials. The load cell is used as feedback information, making the system a closed-loop one. IMUs contain gyroscopes and accelerometers, which are important sensors for human gait information collection [23]. The connection between load cell and wraps is realized by elastic cable which is used to prevent sudden changes of force from causing discomfort to the wearers. Since the whole system is structured mainly with flexible textile materials, it is less restrictive to wearers [21], which makes it not only suitable for healthy people, but also for some patients who require rehabilitation exercises. The weight of the entire system is about 1.8 kg and it is the lightest of all currently known lower limb powered assisted soft exosuit, 84% of the weight is concentrated on the waist of wearer, and the rest is on the thighs. The mass distribution of the lightweight soft exosuit is shown in Table 1.

### 2.2. Stiffness Model

The soft exosuit is mainly composed of textiles and Bowden cables, which makes it difficult to build a kinematic model and control the soft exosuit. In order to reduce the disturbance in the control process, we built a stiffness model of soft exosuit based on the relationship between the angle of hip joint and the length of Bowden cable.

**Figure 2:**
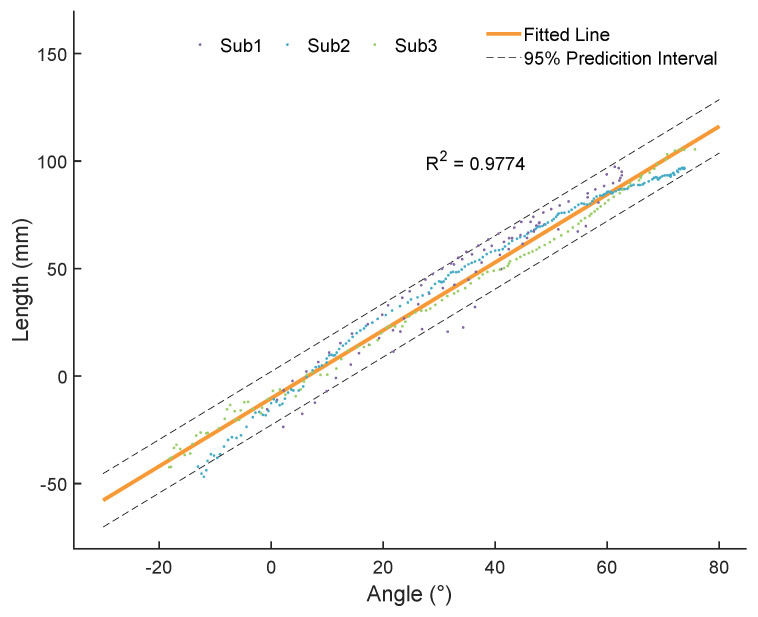
The stiffness model of the soft exosuit. The stiffness model represents the relationship between the angle of hip joint and the length of Bowden cable. The angle of 0 is the angle of hip joint when standing. The positive and negative angles represent the hip flexion and hip extension respectively. The $R^{2}$ is the coefficient of determination.

The movement of the lower limbs can be regarded as a rotation in the sagittal plane during walking. We utilized IMUs and motor encoders to record the angle and the length respectively. Then, three healthy male subjects (174 ± 6 cm, 68 ± 7 kg, 25 ± 2 years old) were invited to do this experiment. Finally, the results of stiffness are shown in the Figure 2.

### 2.3. Human Gait Analysis

As a wearable device dominated by human movement, the lower limb soft exosuit needs to analyze the human gait [22], because the structure of soft exosuit can not interfere with the active movement of humans, and similarly, the period of control strategy is often related to the gait periods, otherwise interference is easily caused.

**Figure 3:**
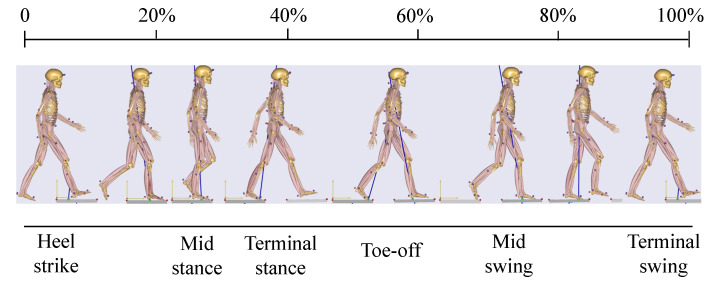
Human Gait Cycle Analysis.

As for walking, human gait generally has a movement pattern [24,25]. The main postures in a whole gait cycle are shown in Figure 3, which includes heel strike, mid-stance, terminal stance, toe-off, mid swing and terminal swing [13,16]. The beginning of gait cycle is at the maximum of right leg hip angle, and the right leg is stand phase and the other leg is swing phase in 20%–60% gait cycle, the left leg is a stand phase while the right leg is a swing phase in 60%–100% [13,16], finally return to the initial state and repeat a new gait cycle [26]. There are many ways to collect gait information, such as vision sensors or IMUs. Chaparro-Rico et al. proposed SANE system to measure the spatiotemporal gait parameters [27], but in this paper, all the gait information data were obtained using IMUs.

## 3. Control

Different joints produce different biological joint torque during walking. In this paper, the lower limbs lightweight soft exosuit was designed to assist hip flexion. Therefore, only the biological torque of hip joint needed to be utilized to control the lightweight soft exosuit. This section focuses on the biological torque analysis of the hip joint during walking on the ground, to develop appropriate control strategies for the lightweight soft exosuit, so as to reduce the metabolic consumption and improve the comfort of the users.

### 3.1. Assistance Force

The control strategy of the lightweight soft exosuit is mainly to change the amplitude and phase of the force [28]. As shown in Figure 3, the heel strike of right leg represents the maximum positive rotation angle of hip, and the toe-off of right leg represents the maximum negative rotation angle of hip. Similarly, the definition of positive and negative direction is also applicable to the left leg. The relatively easy control strategy is to provide a consistent force (about 50–100 N), simultaneously the executive force time starts from the maximum rotation angle of the hip joint in the positive direction to the end of the maximum rotation angle in the negative direction, which strategy is relatively simple and only needs to detect the changing conditions to provide the force. In some papers, many complex functions [29] are used as the function of the force, some of which are even difficult to express with mathematical formulas.

**Figure 4:**
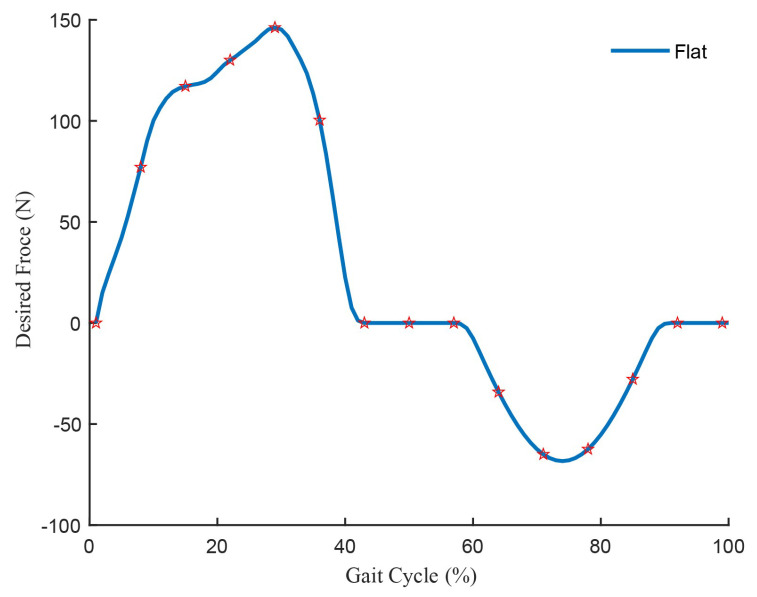
Human hip joint biological torque curve when walking on the ground.

Low limb joint torque, stiffness and range of motion are not only important parameters for analyzing the physical condition of stroke patients [30], but also can be used to analyze the motion information of healthy people. The joints of human body lower limbs will generate torque during walking, which can be referred to as the desired force. Our team used the Vicon (a device for capturing motion) to capture the motion state of the human body’s lower limbs, and utilizes the human body model in Anybody (a kind of human motion simulation software, Denmark) to calculate the angle and torque of each lower limb joint in real time [29]. The biological torque of hip joint when walking on the ground is shown in Figure 4, where it can be discovered that in the first 1/2 cycle, which was a suitable assisting time, the biological torque of hip joint was similar to a sine curve. Because the value of each point was difficult to determine, it was a sine curve instead of the biological torque of hip joint, which is regarded as desired force.

**Figure 5:**
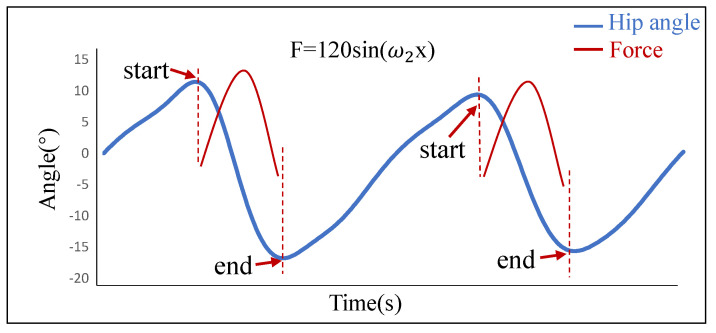
The desired force of the lightweight soft exosuit.

In this paper, the desired force of the lightweight soft exosuit was designed by the biological torque of the human hip joint. In order to prevent the joint damage caused by too much assistance force, the actual desired force was only 80% of the theoretical value. As shown in Figure 5, take 1/2 period of sine function as the desired force, and the executive force time started from the maximum rotation angle of the hip joint in the positive direction to the end of the maximum rotation angle in the negative direction. This is because the impulse, which was produced by the sine force, was less than produced by the consistent force at the same time. Therefore, sine desired force could reduce more impact of the lightweight soft exosuit on the human body, and make users more comfortable.

### 3.2. Control Strategy

**Figure 6:**
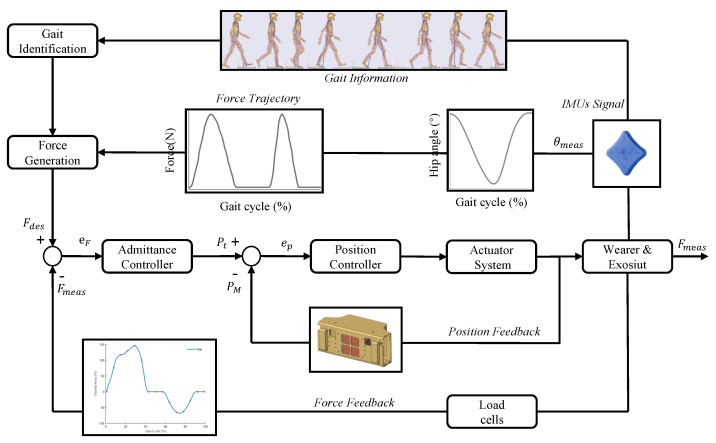
The lightweight soft exosuit control system. $F_{des}$: desired force, $F_{meas}$: measured force, $e_{F}$: force error, $P_{t}$: target position of the motor, $P_{M}$: measured position, $e_{p}$: position error, $\theta_{meas}$: measured angle.

Cao et al. have proposed a hierarchical control strategy based on iterative learning [20]. Our work simplified the hierarchical control strategy and reduced the control complexity. As shown in Figure 6, the control system included an admittance controller, position controller, actuator system, wearer, exosiut, load cells, IMUs, gait identification and force generation. Admittance control has shown good performance in single motion assistance of soft hip exoskeleton [31], and proportional-derivative (PD) controller is used as admittance controller, which can be expressed by Equation (1)

(1) $$P_{t}=K_{p}e_{F}+K_{d}\Delta e_{F}$$

where $K_{p}$ϵ$R^{2\times 2}$ and $K_{d}$ϵ$R^{2\times 2}$ denote respectively the coefficient matrices of the proportional and derivative controllers [29].

When the whole system started running normally, firstly, it obtained the Euler angle of the human body through IMUs, to analyze human gait information. Then according to the gait cycle, the execution time of the assisting force and the desired force ($F_{des}$) were generated. Finally, the admittance controller adjusted the system error, so that the motor could output accurate desired force, and act on the human body, to help users reduce metabolic consumption. Simultaneously, the system also reduced error according to the force feedback and the motor position feedback.

## 4. Evaluation Experiments

In this section, we discuss the experimental results of our lightweight soft exosuit. We utilized the gas analysis equipment (MasterScreen PFT System, Gerge, Germany) and sEMG (Surface Electromyography) acquisition equipment to evaluate the performance of lightweight soft exosuit. It could prove the advantages of the lightweight soft exosuit in reducing metabolic consumption by comparing with other soft exosuit.

### 4.1. Experimental Setup and Protocol

In total, five male and one female college student participated in this experiment. The experiment was approved by the Medical Ethics Committee of Shenzhen Institute of Advanced Technology ((SIAT)-IRB-200715-H0512 (valid time from 2020.01 to 2022.12)), and the content of the experiment and its possible impact were explained to all the participants before conducting the experiment. None of the subjects reported any history of medical illness or psychotropic medication and any medication that would affect the cardiovascular, respiratory, or central nervous system, the specific physical conditions of the subjects [32] are shown in the Table 2.

### 4.2. Metabolic Consumption Experiment

**Figure 7:**
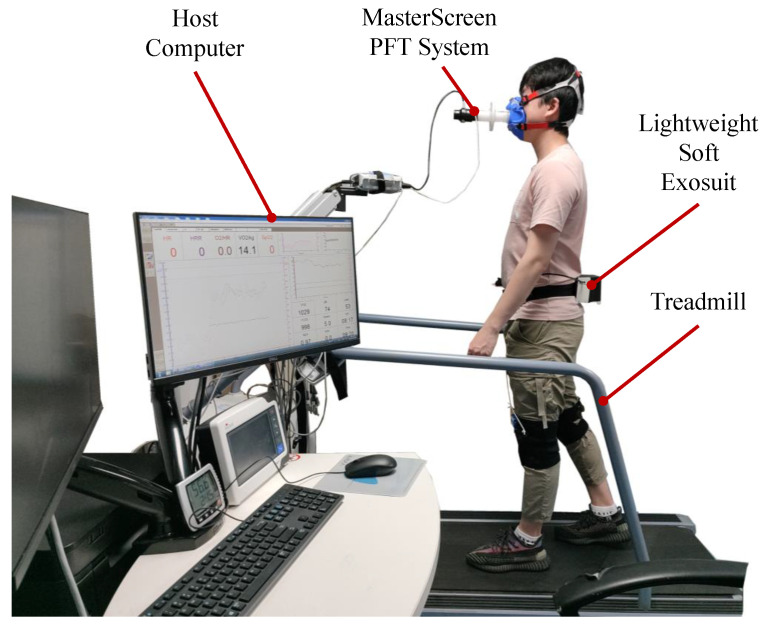
Evaluation experiment of lightweight soft exosuit with MasterScreen PFT System. Subjects wear the lightweight soft exosuit and measure metabolic consumption.

As shown in Figure 7, the whole experimental equipment consisted of a host computer, MasterScreen PFT System, treadmill and lightweight soft exosuit. The subject wore a lower limb lightweight soft exosuit on a treadmill (the treadmill speed sets 5 km/h), which used MasterScreen PFT System to collect metabolic data and do evaluation experiments.

Not only the lightweight soft exosuit, but also the hip flexion assisted soft exosuit (the weight of HFA was about 2.5 kg) and the hip extension assisted soft exosuit (the weight of HEA was about 2.5 kg) which was designed by our team previously, were used to do metabolic consumption experiments. The entire metabolism test experiment consisted of three parts. Each part used a different soft exosuit to do experiments. Firstly, three metabolic tests of the lightweight soft exosuit needed to be completed, which include without using the exosuit, wearing the exosuit without power and wearing the exosuit with power respectively. Similarly, the metabolic consumption tests of hip flexion assisted (HFA) and hip extension assisted (HEA) soft exosuit, needed to be completed without using the soft exosuit, wear the soft exosuit without power and wear the soft exosuit with power respectively. Therefore, in the whole metabolic consumption test experiment, each volunteer needed to finish nine metabolic consumption tests. Each test lasted for 15 min (stand for 3 min and walk normally for 12 min) and had a 10-min rest time after the end. In order to prevent the subjects from completing too many experiments in 1 day and resulting in inaccurate results, only three tests would be completed in one day (three tests for one type of soft exosuit).

The metabolic is computed through the rate of oxygen consumption (*VO*_2_) and the carbon dioxide emission (*VCO*_2_) according to the Brockway equation [33]:

(2) $$\Delta H=c_{1}VO_{{}_{2}}+c_{2}VCO_{{}_{2}}$$

where the ΔH(cal/s) is the energy consumption rate, coefficients $c_{1}$ and $c_{2}$ are 16.89 and 4.84, respectively, and *VO*_2_ and *VCO*_2_ are measured through MasterScreen PFT System.

**Figure 8:**
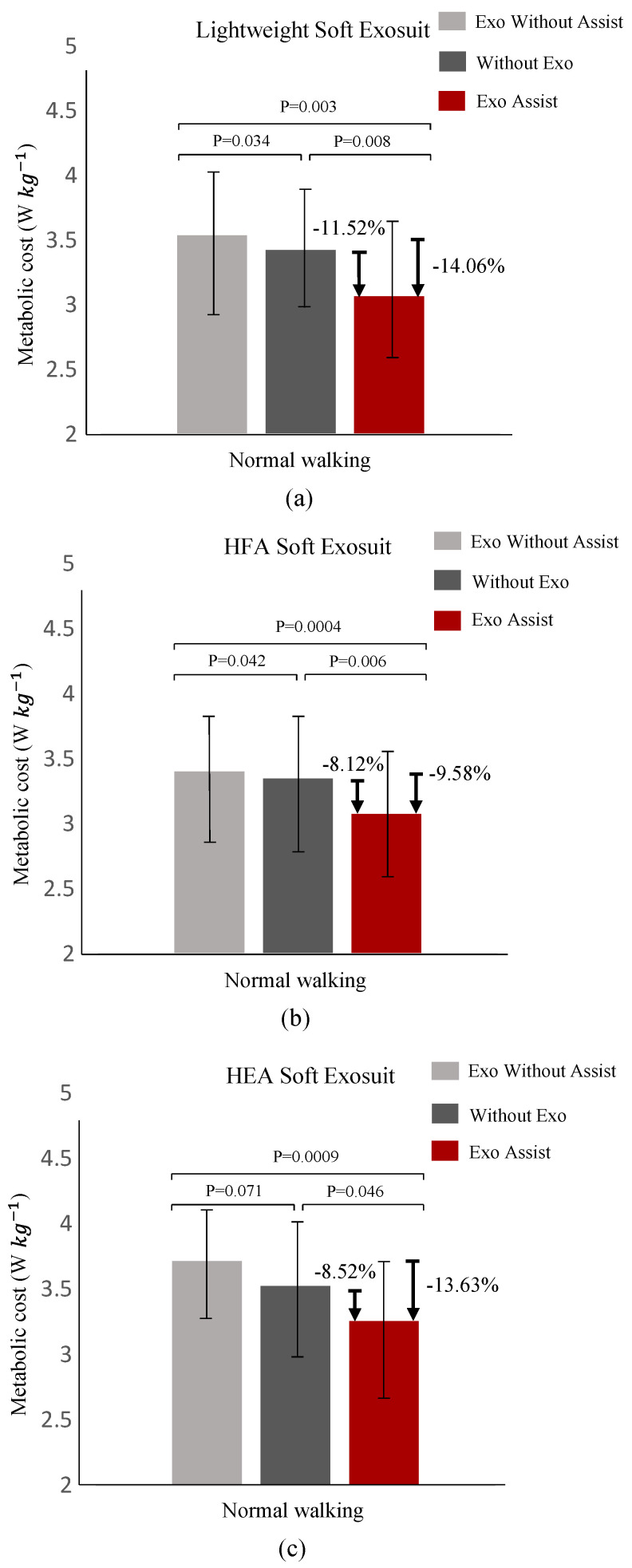
Metabolic consumption which uses MasterScreen PFT System to obtain. (**a**) Using the lightweight soft exosuit to do metabolic consumption experiments through wearing the exosuit without power, without using the exosuit and wearing the exosuit with power respectively. (**b**) Similar to (**a**), using the HFA soft exosuit to do metabolic consumption experiments. (**c**) Similar to (**a**), using the HEA soft exosuit to do metabolic consumption experiments.

As shown in Figure 8, Exo Without Assist, Without Exo and Exo Assist represent wearing the exosuit without power, without using the exosuit and wearing the exosuit with power respectively. From Figure 8a, it can be found that compared with wearing the exosuit without power, the metabolic consumption of using the lightweight soft exosuit was reduced by about 14.06%, and compared with without using the exosuit, it also ccould be reduced by 11.52%. As shown in Figure 8b, compared with wearing the exosuit without power, the metabolic consumption of using the HFA soft exosuit was reduced by about 9.58%, and compared with without using the exosuit, it also could be reduced by 8.12%. Similarly, it also can be discovered that compared with wearing the exosuit without power and without using the exosuit, the metabolic consumption of using the HEA soft exosuit was reduced by about 13.6% and 8.52%, respectively. For the lightweight soft exosuit, it could reduce more metabolic consumption than HFA and HEA soft exosuit, especially in the comparison between without using the exosuit and wearing the exosuit with power. For wearing the exosuit without power and without using the exosuit (in this case, the soft exosuits were only regarded as external load), it can be found that compared with HEA soft exosuit, the lightweight soft exosuit could increase less metabolic consumption, which shows that the weight of lightweight soft exosuit caused less burden and made people more comfortable. Wearing extra equipment brought more metabolic consumption and discomfort to people, therefore, while maintaining the output of the system, decreasing the weight of the system reduced more metabolic consumption and brought comfort to users.

### 4.3. Muscle Fatigue Experiment

**Figure 9:**
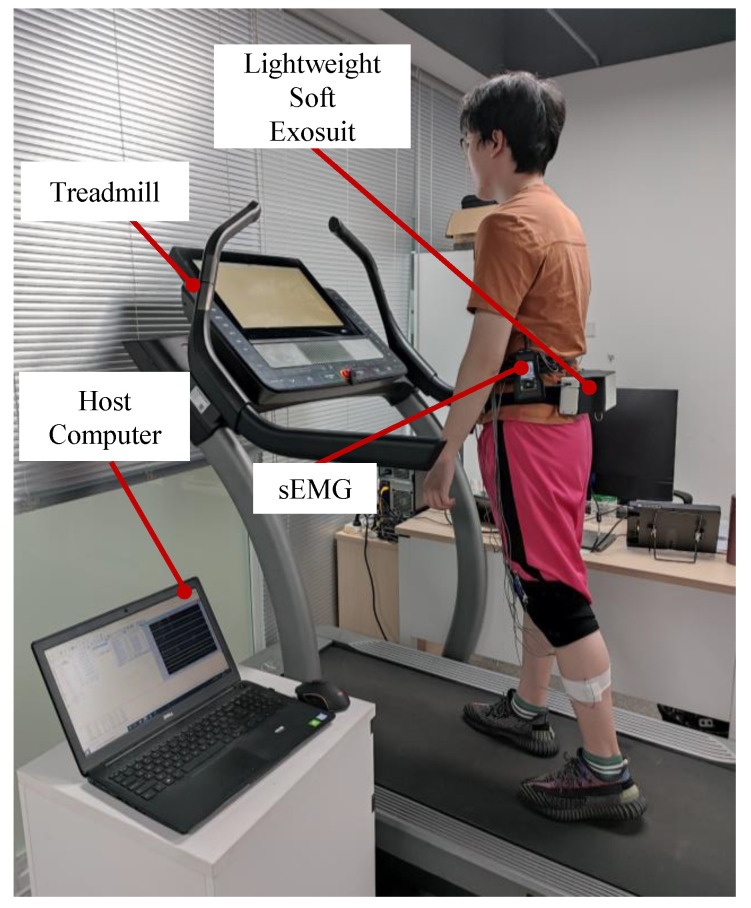
Evaluation experiment of the lightweight soft exosuit with sEMG device. Subjects wear the lightweight soft exosuit and measure muscle fatigue degree.

In the muscle fatigue test experiment, sEMG (the sampling frequency was 2000 Hz) was used to collect sEMG signals and analyze muscle fatigue. As shown in Figure 9, the whole experimental equipment consists of a host computer, sEMG, treadmill and lightweight soft exosuit. The subject wore a lower limb lightweight soft exosuit on a treadmill (the treadmill speed sets 5 km/h), and the sEMG electrode was attached to the left leg muscle, including rectus femoris, vastus lateralis and gastrocnemius.

It was similar to the metabolic consumption experiment. Three muscle fatigue tests of the lightweight soft exosuit needed to be completed, which included initial muscle fatigue before testing, without using the exosuit and wearing the exosuit with power respectively. Therefore, during the entire muscle fatigue test experiment, each subject needed to complete three tests, and each test lasted 6 min and had 10-minutes rest time after the end. Because the experiment was not intense, the subjects would complete all the muscle fatigue experiments on the same day.

**Figure 10:**
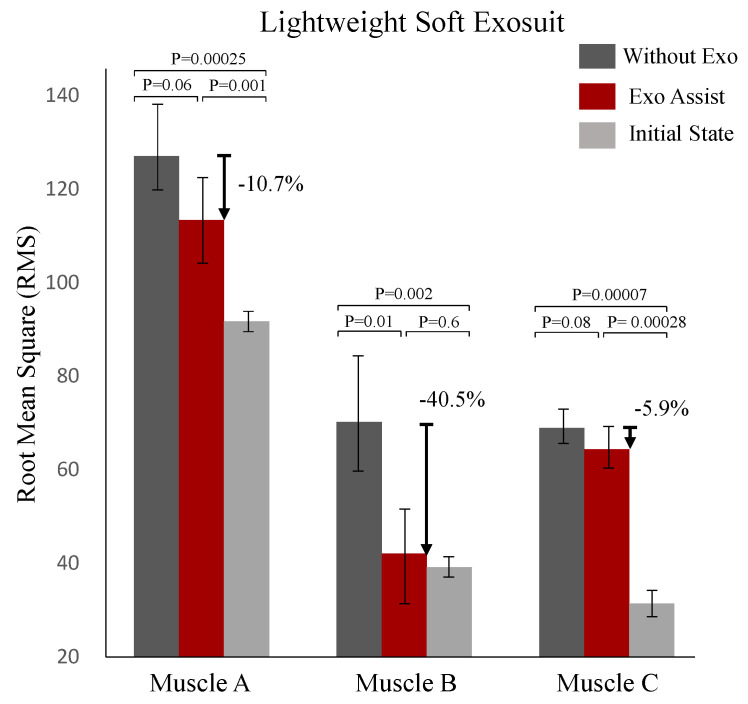
Muscle fatigue degree which uses sEMG to obtain. Muscle A, B and C represent the rectus femoris, vastus lateralis and gastrocnemius, respectively. Exo Assist and Without Exo represent using the lightweight soft exosuit to do muscle fatigue degree experiments through wearing the exosuit with power and without using the exosuit respectively. Initial State represents the initial muscle fatigue degree before testing.

After that, the results of using sEMG to measure human muscle fatigue on a treadmill are shown in Figure 10, which was generated by the lightweight soft exosuit. In this experiment, the rectus femoris, vastus lateralis and gastrocnemius which are commonly used for normal walking [34], were used as experimental muscles. Experimental results used root mean square (RMS) as the evaluation indicator of muscle fatigue degree:

(3) $$EMG_{RMS}\left(n\right)=\sqrt{\frac{1}{n}\sum_{i=n-N+1}^{n}sEMG{\left(i\right)}^{2}}$$

where sEMG(i) is the experimental data collected by each muscle.

The sEMG signals were processed by the time domain method, which is to calculate the RMS value of the EMG signal, therefore the degree of muscle fatigue could be analyzed, and the higher RMS value meant the higher muscle fatigue degree [35]. As shown in Figure 10, muscle A, B and C represent the rectus femoris, vastus lateralis and gastrocnemius, respectively. It can be found that compared with without using the exosuit, the muscle fatigue degree of using the lightweight soft exosuit was reduced by about 10.7%, 40.5% and 5.9% respectively. Therefore, it could be proved that using the lightweight soft exosuit could also reduce human muscle fatigue.

## 5. Discussion

This work presents a lower limb powered lightweight soft exosuit, which is currently the lightest known. From the perspective of user comfort, reducing the weight of the whole system as much as possible under the condition of keeping the soft exosuit system output power unchanged will be a suitable method. Our team uses the Vicon (a device for capturing motion) to capture the motion state of the human body’s lower limbs, and utilizes the human body model in Anybody (a kind of human motion simulation software, Denmark) to calculate the angle and torque of each lower limb joint. Then refer to the torque of hip joint to develop an appropriate assist force for the system. In our experiments, it has been proven that reducing the weight of the system not only improves the comfort of users, but also reduces metabolic consumption and muscle fatigue.

In exercise, the reduction of human metabolic consumption is inextricably related to the lower limb joint assistance. The comparison between some famous soft exoskeletons is presented in Table 3. The whole system designed by our team is lighter than any existing powered hip assisted soft exosuit in weight [16]. In [16], a soft exosuit that assists hip extension, which analyzes the human motion parameters, can reduce metabolic consumption by 9.3%, and the experimental results in [36] proved that assisted hip flexion could reduce metabolic consumption by an average of 5.9% under unpowered condition. The authors in [37] claimed that multi-joint assistance, which includes the assistance torque for ankle and hip joints, is beneficial for reducing net metabolic rate, demonstrating that multi-joint assistance reduces net metabolic rate by 16.93% during walking compared with the absence of any external force assistance. The drive system proposed in [10] is a multi-joint linkage platform, assisting hip extension, hip flexion and ankle plantar flexion through Bowden cable, which reduces net metabolic consumption rate by 14.6%. Soft exosuit, if only assisting ankle joints, are often very light in weight, the ankle assistance in [13] is an unpowered soft exoskeleton, whose weight is only 0.816–1.006 kg and can reduce the metabolic consumption rate by 7.2 ± 2.6%. Although the unpowered soft exoskeletons proposed in [13] are lighter in weight, but they have no power source, so their metabolic consumption rate is lower than ours. However, this work is lighter than other soft exoskeletons in the whole system’s weight and can reduce metabolic consumption by 14.06%. When designing the soft exosuit experiment platform, many difficulties appeared. For example, Bowden cable is prone to thermal wear at the outlet, so the outlet adopts a more accurate fillet process to replace the chamfering process. When we choose the belt, because the belt stiffness is too low, so the loss will increase. Changing the belt with higher stiffness can solve this problem.

Despite that this idea is novel, significant metabolic benefit and muscle fatigue benefit are achieved. There are some limitations to this work. First of all, the lightweight soft exosuit in the experiment only passed the functional test, without mechanical fatigue test, and did not test the duration of continuous operation of the whole system. The system’s weight can also be reduced by optimizing the structure design. Second, evaluation experiments are carried out only on indoor treadmills; it is expected to be tested outdoors. Finally, the specified peak assist force reduces the output peak assist force due to friction from the soft exosuit.

## 6. Conclusions

This work presents a novel lightweight soft exosuit which is currently the lightest among all known powered exoskeletons, which assists hip flexion. Indicated from the result of the experiment, the novel lightweight soft exosuit reduces the metabolic consumption rate of wearers when walking on the treadmill at 5 km per hour by 11.52% compared with locomotion without the exosuit. Additionally, it can reduce more metabolic consumption than the hip extension assisted (HEA) and hip flexion assisted (HFA) soft exosuit which our team designed previously, which has a large weight. The muscle fatigue experiments show that using the lightweight soft exosuit can also reduce muscle fatigue by about 10.7%, 40.5% and 5.9% for rectus femoris, vastus lateralis and gastrocnemius respectively compared with walking without the exosuit. Therefore, this paper demonstrated that decreasing the weight of soft exosuit while maintaining the output almost unchanged can further reduce metabolic consumption and muscle fatigue, and appropriately improve the users’ comfort. In summary, this study focuses on the human comfort, and reduces the weight of the whole system as much as possible under the condition of keeping the soft exosuit output power unchanged, and provides a research direction for future study on how to improve the human comfort which is provided by the soft exosuit. In the future, we will also focus on human comfort and study how to better improve user comfort and experience when wearing the soft exosuit.

## Figures and Tables

**Table 1:** The mass distribution of the lightweight soft exosuit.

Part	Mass(kg)	Location
Waist belt	0.29	Waist
Actuator	0.214	Waist
Batteries	0.53	Waist
MCU	0.08	Waist
IMUs	0.024	Thigh
Wraps	0.22	Thigh
Load cells	0.05	Thigh
Other component	0.392	Waist

**Table 2:** The physical conditions of subjects.

Subjects	Gender	Height (cm)	Weight (kg)	Age (Years Old)
A	Male	182	75	25
B	Male	165	61	21
C	Female	160	45	25
D	Male	176	68	24
E	Male	165	58	24
F	Male	185	102	21

**Table 3:** Comparison of famous soft exoskeletons.

Research	Assistance Mode	Weight (kg)	Power	Net Metabolic Cost (%)
Kim et al. [16]	Hip extension	5.004	Powerd	9.3
Jim et al. [36]	Hip flexion	∖	Powerd	5.9
Sangjun et al. [37]	Hip extension and flexion and Ankle plantar flexion	5.1	Powerd	16.93
Ding et al. [10]	Hip extension and flexion and Ankle plantar flexion	∖	Physiological	14.6
Collins et al. [13]	Ankle plantar flexion	0.816–1.006	Unpowered	7.2 ± 2.6
This work	Hip extension	1.8	Powered	14.06

## Data Availability

The data that support the findings of this study are available on request from the corresponding author.

## Abbreviations

The following abbreviations are used in this manuscript:

IMU	Inertial Measurement Unit
STM32	STMicroelectronics 32-bit Series Microcontroller Chip
PD	Proportional-Derivative
HFA	Hip Flexion Assisted
HEA	Hip Extension Assisted
sEMG	Surface Electromyography
RMS	Root Mean Square
